# Does childhood trauma impact daily psychobiological stress in somatic symptom disorder? An ambulatory assessment study

**DOI:** 10.3389/fpsyt.2022.954051

**Published:** 2022-09-07

**Authors:** Susanne Fischer, Nida Ali, Anja C. Feneberg, Ricarda Mewes, Urs M. Nater

**Affiliations:** ^1^Clinical Psychology and Psychotherapy, Institute of Psychology, University of Zurich, Zurich, Switzerland; ^2^Clinical Psychology, Department of Psychology, University of Vienna, Vienna, Austria; ^3^University Research Platform the Stress of Life (SOLE) – Processes and Mechanisms Underlying Everyday Life Stress, Vienna, Austria; ^4^Outpatient Unit for Research, Teaching and Practice Department of Psychology, University of Vienna, Vienna, Austria

**Keywords:** alpha-amylase, childhood trauma, cortisol, depression, somatic symptom disorder, stress

## Abstract

**Objectives:**

Somatic symptom disorder is characterized by excessive thoughts, feelings, and behaviors dedicated to bodily symptoms, which are often medically unexplained. Although 13% of the population are affected by this disorder, its aetiopathogenesis is not fully understood. Research in medically unexplained conditions (e.g., fibromyalgia) points to increased psychosocial stress and alterations in stress-responsive bodily systems as a potential contributing factor. This pattern has often been hypothesized to originate from early life stress, such as childhood trauma. The aim of this study was to examine, for the first time, whether individuals with somatic symptom disorder exhibit elevated levels of self-reported daily stress and alterations in the autonomic nervous system and hypothalamic-pituitary-adrenal axis, both in comparison to healthy controls and individuals with depressive disorders, and whether reports of childhood trauma influence these alterations.

**Methods:**

A total of *N* = 78 individuals were recruited into this study. Of these, *n* = 27 had a somatic symptom disorder, *n* = 23 were healthy controls, and *n* = 28 had a depressive disorder. All individuals underwent a 14-day measurement period at home, with five assessments of self-reported stress, salivary alpha-amylase, and cortisol per day. Childhood trauma was assessed by the Childhood Trauma Questionnaire.

**Results:**

Individuals with somatic symptom disorder exhibited higher daily stress levels (*p* = 0.063) as well as a less pronounced alpha-amylase awakening response (*p* = 0.050), compared to healthy controls (statistical trends). Moreover, they were characterized by significantly attenuated diurnal cortisol concentrations (*p* < 0.001). A nearly identical pattern was observed in individuals with depression. In individuals with somatic symptom disorder and depressive disorders, childhood trauma was, by trend, associated with a more pronounced alpha-amylase awakening response (*b* = −0.27, *p* = 0.077).

**Conclusions:**

This study provides preliminary evidence for elevated daily stress and blunted sympathetic and hypothalamic-pituitary-adrenal axis activity in individuals with somatic symptom disorder and depressive disorders. Further studies will help to uncover the conditions under which these dysregulations develop into medically unexplained vs. depressive symptoms.

## Introduction

Somatic symptom disorder represents a newly introduced diagnostic category in the fifth edition of the Diagnostic and Statistical Manual of Mental Disorders (DSM-5) (1). It is characterized by excessive thoughts, feelings, and behaviors devoted to bodily symptoms, which can either be part of a somatic disease (e.g., cancer) or “medically unexplained.” Somatic symptom disorder can cause significant distress and impairment. Epidemiological research purports that, currently, it occurs in approximately 13% of the general population (2). However, the aetiopathogenesis of somatic symptom disorder, particularly in the medically unexplained subtype, remains unclear.

Indeed, given the relatively recent introduction of somatic symptom disorder in the DSM-5, only a handful of studies have, as of yet, been dedicated to this subject matter. A number of psychological risk factors have been identified, including self-concept of bodily weakness, catastrophising, and illness-related behaviors, as well as high levels of depression and anxiety (3). By contrast, potential biological correlates of somatic symptom disorder have received less attention. This is unfortunate, as research into other medically unexplained conditions (e.g., fibromyalgia syndrome), has repeatedly attested to the incremental value of a biopsychosocial approach toward these illnesses (4).

One aetiopathogenetic factor that has emerged as particularly relevant in this research is psychosocial stress and alterations in stress-responsive bodily systems (5–7). Concretely, it has been found that extreme forms of stress experienced early in life, such as childhood trauma, are followed by increased levels of everyday life stress in adulthood (8) and can permanently compromise stress-responsive bodily systems, such as the autonomic nervous system and the hypothalamic-pituitary-adrenal axis (9). These findings are in line with models of developmental programming and plasticity, such as the adaptive calibration model (10), as well as the allostatic load model (11), which describe neurobiological alterations in response to environmental demands. Regarding medically unexplained conditions (e.g., fibromyalgia syndrome), a stress-induced pattern of hypocortisolism has been hypothesized, rendering individuals less capable of mounting adequate biological responses to stressors later on in life, potentially facilitating symptoms such as fatigue and pain (12, 13).

Indeed, recent studies on somatic symptom disorder have already shown that this population exhibits higher levels of chronic stress as compared to healthy controls (14). Moreover, somatic symptom disorder appears to be associated with altered autonomic functioning at rest and during emotional processing, such that these individuals exhibit lower parasympathetic activity when contrasted with healthy controls (15). In addition, lower levels of hair cortisol have been reported (14) and lower levels of salivary cortisol in everyday life have been associated with more intense bodily symptoms in somatic symptom disorder (16). However, to date, it remains unknown whether individuals with somatic symptom disorder more frequently experience daily life stress and diurnal changes in autonomic and hypothalamic-pituitary-adrenal axis activity, and whether this is affected by the presence of childhood trauma.

The main aim of the present study was to investigate, for the first time, whether individuals with somatic symptom disorder experience higher levels of self-reported daily stress and alterations in stress-responsive bodily systems. Given that stress is a non-specific risk factor for all kinds of physical and mental illnesses (17), we chose both a healthy and a clinical comparison group. Our clinical comparison group was composed of individuals with depressive disorders, which have been extensively linked to stress, both on a psychological (18, 19) and biological level (20, 21). Concretely, it has been found that individuals with depressive disorders exhibit higher levels of childhood trauma and chronic stress, as well as attenuated sympathetic functioning and higher levels of cortisol. Comparing individuals with depressive disorders with individuals with somatic symptom disorder is particularly interesting given that previously, medically unexplained physical symptoms were often understood as a form of “masked depression” (22) and phenomenological distinctions between the two disorders remain challenging to this day. Identifying specific biological profiles for each disorder could thus aid differential diagnosis.

Our hypotheses were as follows: We expected that (a) both individuals with somatic symptom disorder and depressive disorders would report higher levels of self-reported daily stress when compared to healthy controls. Next, we hypothesized that (b) both groups would differ from the healthy controls regarding their diurnal salivary alpha-amylase activity, a proxy of sympathetic activation (23). We also predicted that (c) individuals with somatic symptom disorder would exhibit lower diurnal salivary cortisol and individuals with depressive disorder would exhibit higher diurnal cortisol when compared to healthy controls. Finally, we wanted to explore whether previous-day self-reported stress would be associated with diurnal alpha-amylase and cortisol.

The second aim of the present study was to investigate to what extent these hypothesized patterns were associated with childhood trauma. We predicted that, in individuals with somatic symptom disorder and depressive disorders, those with childhood trauma would (d) exhibit higher levels of self-reported daily stress and more pronounced alterations in alpha-amylase and cortisol when compared those without childhood trauma.

## Methods

### Participants

This study was part of two larger projects: one on somatic symptom disorder and depressive disorder (24) and one on chronic fatigue, which also included a sample of healthy controls. The sample of the present study is composed of individuals with somatic symptom disorder (*n* = 27) and individuals with depressive disorders (*n* = 28) from project 1 as well as the healthy controls (*n* = 23) from project 2. All participants were recruited from the general population as well as from primary and secondary care. Both studies were conducted at the University of Marburg, Germany, between 2011 and 2016.

The inclusion criteria for the individuals with *somatic symptom disorders* and *depressive disorders* were: fluency in German, age 18 years or above, identifying as female, Body Mass Index (BMI) < 30, and fulfilling either the DSM diagnostic criteria for a somatic symptom disorder or a depressive disorder (but not both; see below). The exclusion criteria were: pregnancy, lactation, major physical diseases (e.g., cancer, hepatic, hematological, neurological, autoimmune, or endocrinological diseases), major mental disorders (i.e., substance abuse/dependence within the past 2 years, eating disorders within the past 5 years, lifetime psychotic or bipolar disorder). The inclusion criteria for the *healthy controls* were: fluency in German, age 18 years or above, identifying as female, BMI <30. The exclusion criteria were: physical and mental ill health, intake of medication.

Although the sample sizes of the three groups were not calculated for the purpose of the present study, the number of recruited individuals is similar to previous research on cortisol in individuals with somatic symptom disorder or its predecessor category, somatoform disorders (14, 25) and was large enough to detect large-sized effects according to a G^*^Power analysis (α = 0.05, 1-β = 0.80).

### Procedures

All study relevant procedures were identical for the two projects and all investigations were carried out in accordance with the latest version of the Declaration of Helsinki. The study protocols were approved by the local Ethics Committee (University of Marburg) and written informed consent was obtained from all participants.

### Telephone interview

All participants were screened for eligibility via telephone. Somatic symptom disorder was diagnosed according to DSM-5 criteria (1), using a structured interview that was used in several prior studies (26, 27). Importantly, the participants' symptoms had to be medically unexplained, as otherwise, any pathophysiological findings would potentially be confounded by the presence of (a) somatic disease(s) (e.g., cancer). Depressive disorders were diagnosed according to DSM-IV criteria using the Structured Clinical Interview for DSM (SCID) (28). All interviewers were trained psychologists. A comprehensive medical history was obtained to screen for exclusionary physical diseases.

### Laboratory appointment

Upon fulfilling eligibility criteria, the participants were invited to an in-person appointment at the University of Marburg. First, all participants completed a battery of questionnaires (see below). Second, the participants were instructed in stress reporting and saliva sampling procedures for the 14-day ambulatory assessment period, which was supported by a pre-programmed application on an iPod touch^®^ (G. Mutz, Cologne; see below).

### Ambulatory assessment (14 days)

During the 14-day ambulatory assessment period, the participants activated the application on the iPod touch^®^ each morning, upon awakening. This was followed by pre-programmed alarms 30 min later, and at 11 AM, 2 PM, 6 PM, and 9 PM. At each of these time points, participants typed the number of the respective SaliCap^®^ (IBL, Hamburg, Germany) sampling tube into the iPod, and started to accumulate saliva in their mouths for 2 min before salivating into the SaliCap^®^ via a plastic straw. They then stored the saliva samples in their freezers or refrigerators until the end of the study. From 11 AM onwards, at each time point the participants also answered a question about their current stress level. After completing the ambulatory assessment, the participants were invited to another laboratory appointment to return their iPod touch^®^ and saliva samples, which were immediately stored at −20°C.

## Psychological measures

Childhood trauma was assessed using the German version of the Childhood Trauma Questionnaire (CTQ) (29). This 25-item scale inquires about five types of childhood trauma: emotional abuse, physical abuse, sexual abuse, emotional neglect, and physical neglect. The presence of childhood trauma was assumed if respondents scored at or above specific validated cut-offs for moderate to severe trauma on at least one of the five subscales (10 for emotional abuse, 15 for emotional neglect, and 8 for the remaining categories) (30).

Depression severity was measured with the German version of the Patient Health Questionnaire depression module (PHQ-9) (31), which reflects the nine symptoms of a major depressive episode according to the DSM-5 (1). Adding all items up results in a total score between 0 and 24.

Somatic symptom severity was assessed via the German version of the Patient Health Questionnaire somatic symptom module (PHQ-15) (31). Importantly, only 13 of the original 15 items were used in the present study, as these two (fatigue and sleeping problems) also feature in the PHQ-9. The score range of this shortened version is between 0 and 26.

Stress as occurring in everyday life (“At the moment, I feel stressed”) was measured via a five-point Likert scale ranging from 0 to 4, as done previously (32, 33).

## Biological measures

The analyses of salivary alpha-amylase and cortisol were conducted at the Biochemical Laboratory of the Department of Clinical Biopsychology, University of Marburg. Saliva samples were analyzed using reagents from Roche (Basel, Switzerland) for alpha-amylase, and immunoassays from IBL (Hamburg, Germany) for cortisol. For alpha-amylase the intra-assay coefficient of variation was <10% and the inter-assay coefficient of variation was <13%. For cortisol the intra- and inter-assay variation was ≤ 10%.

### Statistical analysis

#### Data preparation

To check for compliance with the sampling protocol, the time stamps of the mobile application were used. For the awakening and +30 min time points, all alpha-amylase and cortisol samples that deviated more than 20 min from the schedule were excluded (24). For the 11 AM, 2 PM, 6 PM, and 9 PM time points, all stress reports and salivary samples that deviated more than 2 h were excluded. For stress, 3% of the data points were excluded as a result of this. For alpha-amylase and cortisol, 8% data points were excluded. The remaining alpha-amylase and cortisol values were tested for normal distribution and were subsequently log-transformed. The alpha-amylase awakening response (AAR) and the cortisol awakening response (CAR) were calculated by subtracting the value of the first measurement time point from the +30 min measurement time point. The AAR and CAR refer to characteristic changes of alpha-amylase and cortisol in response to awakening and are regarded as markers of the sympathetic nervous system and of the hypothalamic-pituitary-adrenal axis, respectively, reflecting regulatory capacity of these systems (34). To obtain an index of daily self-reported stress, alpha-amylase activity, and cortisol secretion, mean levels of the 11 AM, 2 PM, 6 PM, and 9 PM time points were calculated.

#### Data analysis

To investigate whether the three diagnostic groups differed with respect to age, Body Mass Index (BMI), smoking, somatic symptoms, depressive symptoms, and childhood trauma, Kruskal-Wallis and Mann-Whitney *U* tests were computed. To compare the three groups with respect to self-reported daily stress, AAR, diurnal alpha-amylase, CAR, and diurnal cortisol averaged across the 14 days, univariate ANCOVAs were conducted. Age and the intake of medication were added as control variables (a priori specification) in the models testing the effects of self-reported stress. In the models testing the effects on alpha-amylase and cortisol, age, BMI, smoking status, and intake of medication were controlled for. To examine the effects of previous-day self-reported stress and childhood trauma on daily stress, diurnal alpha-amylase, and diurnal cortisol, multilevel models were calculated in three steps (null model, level 1 predictor model, level 2 predictor model). The final models included previous-day stress as a level 1 predictor and covariates and childhood trauma as level 2 predictors (time-lagged models). The level of statistical significance was set at α = 0.05. Descriptives are reported as mean ± standard deviation for normally distributed variables, median (interquartile range) for non-normally distributed variables, and absolute and relative frequencies for categorical variables. All analyses were conducted in SPSS 28.

## Results

### Participant characteristics

Participant characteristics can be found in Table 1. The median age was 25 (9.25) and the median BMI was 22 (4.7). Nearly a third (27%) of the sample indicated that they were smokers. A Kruskal-Wallis test indicated that the three groups significantly differed in age [H_(2)_ = 9.12, *p* = 0.010]. *Post-hoc* tests adjusted for multiple testing indicated that individuals with somatic symptom disorder (*p* = 0.074) and depressive disorders (*p* = 0.011) were (by trend) younger than the healthy controls. In contrast, the three groups did not differ on their BMI scores [H_(2)_ = 0.50, *p* = 0.78].

**Table 1:** Characteristics of individuals with somatic symptom disorders, depressive disorders, and healthy controls (*N* = 78).

	**Somatic symptom disorder (*n* = 27)**	**Depressive disorder (*n* = 28)**	**Healthy control (*n* = 23)**
Age (years)^I^	25 (8)	24 (4.8)	33 (25)
Body mass index (kg/m^2^)	21.3 (5.5)	21.7 (5.4)	21.7 (2.4)
Smoking (yes)	9 (33%)	10 (36%)	2 (9%)
Somatic symptom severity (PHQ-15)	10 (5)	8 (5.8)	-
Depression severity (PHQ-9)^II^	6 (4)	17 (6.5)	-
Medication (yes)			
Antihypertensives	0	0	-
Analgesics	7 (26%)	1 (4%)	-
Antidepressants	0	4 (14%)	-
Childhood trauma (CTQ)	35.8 ± 9.4	42.5 ± 14.8	40.2 ± 14.2

Medians and interquartile ranges (indicated by brackets), absolute and relative frequencies (indicated by “%”), and means and standard deviations (indicated by “±”) are presented. Group comparisons were conducted using Kruskal-Wallis tests, Mann-Whitney U tests, and univariate ANOVAs.

^I^both individuals with somatic symptom disorder and depressive disorders were younger than healthy controls disorder (p = 0.025 and p = 0.004, respectively).

^II^individuals with depressive disorders had more depressive symptoms than individuals with somatic symptom disorder (p < 0.001).

PHQ-15 = Patient Health Questionnaire – somatic symptom module (13 items rated by frequency of occurrence; score range from 0 to 26).

PHQ-9 = Patient Health Questionnaire – depression module (9 items rated by frequency of occurrence; score range from 0 to 27).

CTQ = Childhood trauma questionnaire (25 items rated by frequency of occurrence; score range from 25 to 125).

As expected, individuals with a somatic symptom disorder had a tendency toward a higher somatic symptom load than individuals with depressive disorders [10 (5) vs. 8 (5.8); *U* = 273.00, *p* = 0.076], whereas individuals with depressive disorders had more depressive symptoms than individuals with a somatic symptom disorder [17 (6.5) vs. 6 (4); *U* = 732.50, *p* < 0.001], according to Mann-Whitney *U* tests.

Over half of the sample (54%) had experienced childhood trauma: 40% reported emotional abuse, 13% reported physical abuse, 10% reported sexual abuse, 24% reported emotional neglect, and 35% reported physical neglect. The three groups did not differ in the amount of trauma experienced [F_(2,73)_ = 1.87, *p* = 0.161].

### Effects of diagnostic group on daily stress

To investigate whether individuals with somatic symptom disorder and depressive disorders had higher self-reported daily stress levels than healthy controls, a univariate ANCOVA was conducted. The results indicated that there was a significant difference in daily stress levels reported by the three groups [F_(2,73)_ = 9.96, *p* < 0.001. partial eta^2^ = 0.21]. According to *post-hoc* testing, individuals with depressive disorders had significantly higher self-reported stress than healthy controls (*p* < 0.001) and individuals with somatic symptom disorder (*p* = 0.006). Individuals with somatic symptom disorder had higher stress levels than healthy controls by trend (*p* = 0.063).

### Effects of diagnostic group on diurnal alpha-amylase

**Figure 1:**
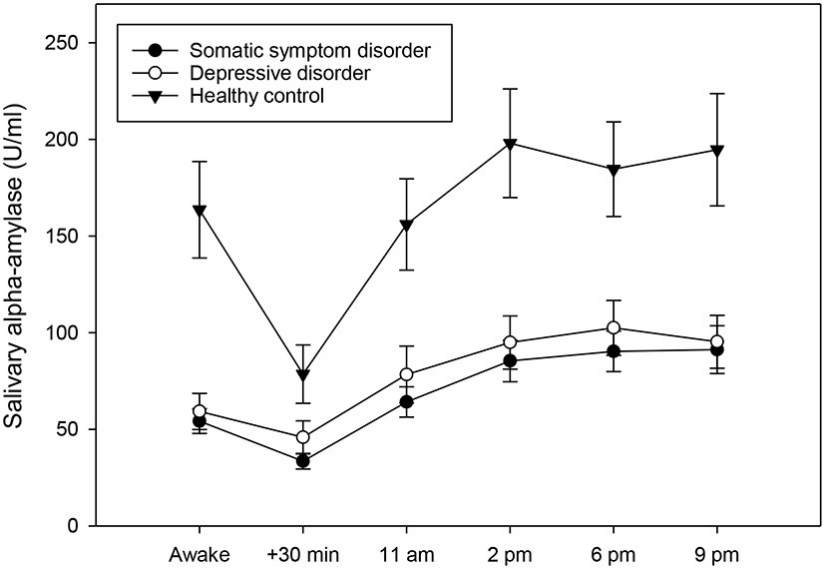
Diurnal profiles of salivary alpha-amylase in individuals with somatic symptom disorder (*n* = 27), individuals with depressive disorders (*n* = 28), and healthy controls (*n* = 23), averaged across 14 days. Values represent means and standard errors. Individuals with somatic symptom disorder and depressive disorders had a less pronounced alpha-amylase awakening response when compared to healthy controls, while there was no group difference in mean diurnal alpha-amylase activity.

To investigate whether individuals with somatic symptom disorder and depressive disorders differed from healthy controls in their diurnal alpha-amylase activity, a univariate ANCOVA was conducted. The analysis revealed that the three groups differed significantly in their AAR [F_(2,68)_ = 3.16, *p* = 0.049, partial eta^2^ = 0.09] (Figure 1). Post-hoc tests indicated that individuals with somatic symptom disorder (*p* = 0.050) and with depressive disorders (*p* = 0.017) had a less pronounced AAR than healthy controls (the former result was only a statistical trend). By contrast, the three groups did not differ in their daily alpha-amylase activity [F_(2,68)_ = 1.59, *p* = 0.307].

### Effects of diagnostic group on diurnal cortisol

**Figure 2:**
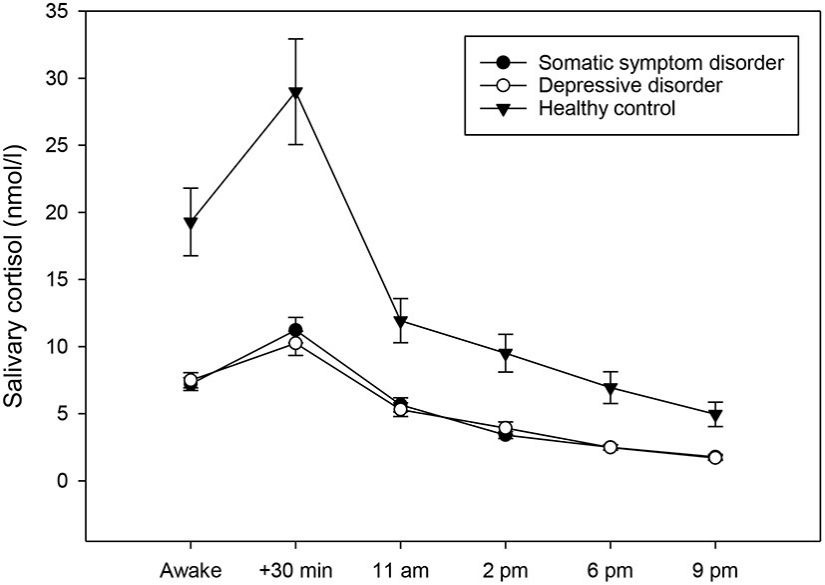
Diurnal profiles of salivary cortisol in individuals with somatic symptom disorder (*n* = 27), individuals with depressive disorders (*n* = 28), and healthy controls (*n* = 23), averaged across 14 days. Values represent means and standard errors. Individuals with somatic symptom disorder and depressive disorders had significantly lower mean diurnal cortisol concentrations when compared to healthy controls, while there was no group difference in the cortisol awakening response.

To test whether individuals with somatic symptom disorder and depressive disorders had attenuated/elevated diurnal cortisol concentrations compared to healthy controls, a univariate ANCOVA was computed. The results showed that the three groups did not exhibit any differences in CAR [F_(2,70)_ = 0.61, *p* = 0.546]. They did, however, vary significantly regarding their diurnal cortisol secretion [F_(2,70)_ = 10.82, *p* < 0.001, partial eta^2^ = 0.24; see also Figure 2]. *Post-hoc* testing revealed that individuals with somatic symptom disorder (*p* < 0.001) and those with depressive disorders (*p* = 0.001) had significantly lower cortisol levels across the day when compared to healthy controls.

### Effects of childhood trauma on daily stress

In order to investigate whether the presence of childhood trauma was associated with the comparably elevated levels of daily stress in individuals with somatic symptom disorder and depressive disorders, a multilevel model was run. According to this model, childhood trauma was not a significant predictor of daily stress (*b* = 0.18, *p* = 0.288).

### Effects of childhood trauma on diurnal alpha-amylase

To examine whether, in individuals with somatic symptom disorder and depressive disorders, previous-day stress and childhood trauma was related to diminished AAR, multilevel models were computed. Childhood trauma had an effect on AAR by trend (*b* = −0.27, *p* = 0.077), such that individuals with a history of childhood trauma had a more pronounced AAR, whereas there was no effect of previous-day stress on the AAR (see Table 2). Age had a direct effect on mean diurnal alpha-amylase activity (*b* = 0.02, *p* = 0.027). No other effects emerged.

**Table 2:** Fixed effects for hierarchical linear models predicting alpha-amylase in individuals with somatic symptom disorder and depressive disorders, using maximum likelihood.

	**Alpha-amylase awakening response**			**Diurnal alpha-amylase**		
	***b***	**SE**	***t***	***b***	**SE**	***t***
Intercept	−0.81	0.58	−1.39	4.56	0.77	5.92****
Level 2						
Age	<0.01	0.01	−0.02	0.02	0.01	2.28**
Body mass index	0.02	0.03	0.89	−0.04	0.04	−1.26
Smoking status	0.21	0.16	1.29	−0.29	0.22	−1.34
Medication intake	0.09	0.19	0.48	−0.35	0.26	−1.35
Childhood trauma^I^	−0.27	0.15	−1.82*	−0.09	0.21	−0.45
Level 1						
Self-reported stress (same day)	0.05	0.07	0.76	< -0.01	0.02	−0.16
Self-reported stress (previous day)	0.02	0.07	0.34	0.01	0.02	0.56

b, estimate; SE, standard error.

*p <0.10; **p < 0.05; ****p < 0.001.

^I^Childhood trauma was assessed by the Childhood Trauma Questionnaire (25 items rated by frequency of occurrence; score range from 25 to 125) and dichotomised according to validated cut-offs for moderate to severe trauma (0 = no trauma, 1 = trauma).

### Effects of childhood trauma on diurnal cortisol

In order to test whether, in individuals with somatic symptom disorder and depressive disorders, previous-day stress and childhood trauma predicted attenuated diurnal cortisol, multilevel models were performed. The variance in the CAR was not high enough to conduct multilevel modeling. Neither previous-day stress nor childhood trauma had an effect on mean diurnal cortisol, but BMI did (*b* = −0.06, *p* = 0.005).

## Discussion

The main aim of the present study was to investigate, for the first time, whether individuals with somatic symptom disorder exhibit elevated levels of self-reported daily stress and alterations in stress-responsive bodily systems. We found that both individuals with somatic symptom disorder and individuals with depressive disorders tended to report more stress over the course of a 14-day period when compared healthy controls. We also found that the two groups had a less pronounced AAR and significantly lower diurnal cortisol concentrations. The second aim of this study was to investigate whether these findings were associated with childhood trauma. Here, we found that in individuals with somatic symptom disorder and depressive disorders, those reporting childhood trauma tended to have a more pronounced AAR.

The finding that individuals with somatic symptom disorder tended to report higher levels of daily stress than healthy controls, is in line with the notion of stress being a key aetiopathogenetic factor in medically unexplained conditions, such as chronic fatigue syndrome, fibromyalgia syndrome, or irritable bowel syndrome (5–7). Our study extends prior research by being the first to be conducted in this patient population, for which, due to its relatively recent introduction in the DSM-5 (1), little knowledge on risk or maintaining factors is available. Our results are in line with a recent finding demonstrating elevated chronic levels of stress in this group (14) and complements it by employing a more unbiased approach to measuring stress, with repeated assessments and high ecological validity due to everyday life assessments. Notably, individuals with depressive disorders experienced even higher levels of daily stress than individuals with somatic symptom disorder, which is in line with prior research in this population (18) and implies that everyday life stress is a non-specific correlate of certain mental disorders.

The finding that individuals with somatic symptom disorder had a tendency toward a less pronounced AAR than healthy controls, adds to prior research demonstrating reduced parasympathetic functioning in this population during the processing of emotional stimuli (15). In the same study, salivary alpha-amylase activity did not distinguish individuals with somatic symptom disorder from healthy controls. This could mean that, whereas sympathetic regulation is not generally altered in somatic symptom disorder, it may be compromised once the system is challenged, as is the case during awakening. Although research into alpha-amylase as a biomarker of stress has been ongoing for nearly three decades (35), the consequences of altered awakening responses for the individual are still not fully understood. A recent study has suggested a link between blunted AAR and impaired work performance (36). Further investigations will be necessary to unravel its implications in somatic symptom disorder (e.g., regarding general arousal and cognitive functioning). Regarding cortisol, markedly reduced diurnal concentrations were found in individuals with somatic symptom disorder. This finding corroborates the results of a prior study by our group in which hair cortisol, a cumulative marker of three-month endocrine secretion, was found to be reduced in the same group of individuals (14). It resonates well with the long-standing notion of hypocortisolism in medically unexplained conditions, which could facilitate fatigue and pain by means of inflammatory disinhibition (12, 13). Again, neither of these findings appear to be specific to somatic symptom disorder, as individuals with depressive disorders exhibited the same patterns. In major depressive disorder, the general notion is that cortisol is elevated (20). However, atypical vs. melancholic depression appears to be associated with relatively lower levels of cortisol. Moreover, anxious depression has been associated with hypocortisolism (37), which could explain the present findings. Future research with large samples that allow for further subtyping will be necessary to illuminate this issue.

Our finding that childhood trauma was unrelated to self-reported stress as assessed in daily life contradicts previous research in healthy adults, which has shown programming effects of early life stress. For instance, a large-scale population-based study has found that individuals with adverse childhood experiences had a higher risk of experiencing everyday life stress in adulthood (8). Notably, this study employed a retrospective measure of daily stress. It is thus possible that the link between childhood trauma and daily stress was influenced by the methodology. Regarding biological outcomes, a meta-analysis recently provided evidence for “traumatic effects” on the autonomic nervous system and the hypothalamic-pituitary-adrenal axis (9). This study examined cardiovascular reactivity to psychological stress in the laboratory in healthy individuals, and found blunted responses in association with childhood trauma. We complement these findings by providing tentative evidence for a more pronounced AAR in traumatized individuals. Another meta-analysis focusing on cortisol has not found consistent effects of childhood trauma on the CAR and on resting cortisol (38), which is in line with the herein reported null-findings regarding these parameters. Notably, the overall level of childhood trauma was relatively low in the present sample and the three groups did not differ in their degree of trauma. This is at odds with the majority of previous research [see (5–7, 19) for reviews/meta-analyses]. One explanation for this finding might be that, whereas prior studies often recruited patients from secondary or tertiary health care settings (e.g., clinics), our sample was mainly composed of individuals from the general population. Another contributing factor may be the time-consuming 14-day study design, which may have been too stressful for highly stress sensitive individuals such as those with high levels of trauma and psychopathology.

The present study comes with a number of strengths. First, we provide data on daily stress levels, salivary alpha-amylase, and cortisol, which is unprecedented in individuals with somatic symptom disorder. Further strengths include a carefully screened sample of individuals, which allowed us to eliminate a number of important confounders (e.g., comorbid somatic and mental illnesses). Moreover, a 14-day measurement period was employed and measures were taken in individuals' daily lives, which maximizes the reliability and ecological validity of our findings, respectively. However, this study also has some limitations. First, due to the careful screening, our overall sample size (*N* = 78) was relatively small, and replications of our findings in larger cohorts are called for. Second, only individuals identifying as women were included, which means that our findings cannot be generalized to individuals of other genders. Related to this, we did not control for menstrual cycle phases, which might have influenced our biological measures. Third, since there was no a priori matching of individuals with somatic symptom disorder/depression with healthy controls, there was a significant age difference between our groups, which could have impacted on our findings. However, age was included as a covariate in all statistical models and was not found to be related to daily stress, AAR, or diurnal cortisol. Fourth, although in general, our sampling schedule was rather extensive, the AAR and CAR were only captured by means of two time points (awakening and +30 min). It is thus possible that further group differences could have emerged if we had applied even more nuanced samplings. Fifth, our research question necessitated the exclusion of comorbid major mental disorders, such as anxiety disorders. However, since comorbidities are a frequent occurrence in clinical practice it is important for future research to investigate stress in individuals with comorbid somatic symptom disorder. Finally, some of the individuals in the somatic symptom disorder and depressive disorders groups were on medication, which could have impacted the biological measures. However, given that this only applied to a minority of participants, and that medication intake was included as a confounder, it is unlikely that this had a significant effect on our findings.

In sum, this study provides initial evidence for elevated daily stress and blunted sympathetic and hypothalamic-pituitary-adrenal axis activity in individuals with somatic symptom disorder. It also shows that childhood trauma exerted potential effects on the AAR. Further studies will help to uncover the conditions under which these dysregulations develop into medically unexplained vs. depressive symptoms.

## Data availability statement

The raw data supporting the conclusions of this article will be made available by the authors, without undue reservation.

## Ethics statement

The studies involving human participants were reviewed and approved by the local Ethics Committee (University of Marburg). The patients/participants provided their written informed consent to participate in this study.

## Author contributions

RM and UN conceived and designed the studies. NA and AF prepared the data and SF analyzed, interpreted the data and drafted the article. NA, AF, RM, and UN revised it critically for important intellectual content. All authors have approved the final article.

## Funding

This research was supported by the Volkswagen foundation (AZ.:II/84905) and by the University of Marburg.

## Conflict of interest

The authors declare that the research was conducted in the absence of any commercial or financial relationships that could be construed as a potential conflict of interest.

## Publisher's note

All claims expressed in this article are solely those of the authors and do not necessarily represent those of their affiliated organizations, or those of the publisher, the editors and the reviewers. Any product that may be evaluated in this article, or claim that may be made by its manufacturer, is not guaranteed or endorsed by the publisher.
